# Preterm Birth, Intrauterine Infection, and Fetal Inflammation

**DOI:** 10.3389/fimmu.2014.00574

**Published:** 2014-12-01

**Authors:** Matthew W. Kemp

**Affiliations:** ^1^School of Women’s and Infants’ Health, The University of Western Australia, Perth, WA, Australia

**Keywords:** preterm birth, fetus, inflammation, infection, injury

## Abstract

Preterm birth (PTB) (delivery before 37 weeks’ gestation) is a leading cause of neonatal death and disease in industrialized and developing countries alike. Infection (most notably in high-risk deliveries occurring before 28 weeks’ gestation) is hypothesized to initiate an intrauterine inflammatory response that plays a key role in the premature initiation of labor as well as a host of the pathologies associated with prematurity. As such, a better understanding of intrauterine inflammation in pregnancy is critical to our understanding of preterm labor and fetal injury, as well as on-going efforts to prevent PTB. Focusing on the fetal innate immune system responses to intrauterine infection, the present paper will review clinical and experimental studies to discuss the capacity for a fetal contribution to the intrauterine inflammation associated with PTB. Evidence from experimental studies to suggest that the fetus has the capacity to elicit a pro-inflammatory response to intrauterine infection is highlighted, with reference to the contribution of the lung, skin, and gastrointestinal tract. The paper will conclude that pathological intrauterine inflammation is a complex process that is modified by multiple factors including time, type of agonist, host genetics, and tissue.

## Introduction

Preterm birth (PTB) is presently defined as delivery before 37 weeks’ completed gestation from the last known menstrual period (1); the lower limit of PTB varies between countries but is generally set at 20 or 22 weeks’ completed gestation (2). PTB is often further sub-classified into late (32 or 34–36 completed weeks’ gestation), early (<32 completed weeks’ gestation), and very early (<28 completed weeks’ gestation) delivery (2, 3). These definitions are now viewed as somewhat arbitrary in nature and susceptible to classification bias because of the methodologies used to assess gestational age (self-reported last menstrual period vs. ultrasound measurement) (2, 4, 5). These measures also fail to account for a host of pregnancy characteristics (e.g., infection, maternal health status, and fetal distress) that are likely key to both the treatment of the preterm infant and our attempts to better understand and prevent PTB. As such, a number of investigators have recommended the adoption of an information-rich, phenotypic PTB classification system that considers births occurring after 16 and before 39 weeks of completed gestation (5).

Preterm birth is a significant global health issue. Rates of PTB vary with ethnicity, geography, and a range of lifestyle factors (6); in 2003, the rate of PTB for Asian, Caucasian, and African American women in the United States was 10.5, 11.5, and 17.8%, respectively (7). Complications of prematurity account for 29% of global neonatal deaths (approximately 1 million) each year and 3.1% of total disability adjusted life years in the global burden of disease (8, 9). Underscoring the scope of the PTB problem, recent data suggest that just a 5% relative reduction (from 9.59 to 9.07%) in the rate of PTB across 39 countries with a very high-human development index would yield some 58,000 fewer preterm deliveries and a saving of US$3 billion (10).

From a pathophysiological perspective, PTB is a highly complex and incompletely understood syndrome (11, 12). Evidence exists to implicate a host of factors including uteroplacental ischemia, cervical disease, decidual hemorrhage, stress, infection, and inflammation in the initiation of prematurity (12). Inflammation (with or without hypoxia) is also strongly implicated in a host of fetal morbidities. It is hypothesized that these, and perhaps other factors, acting either independently or in concert, trigger a suite of changes in gestational tissues that shift the uterus from a state of quiescence to one of activity in the initiation of labor. Gotsch and colleagues have described the tripartite uterine components of a (p. 6) “common pathway of parturition” as including an increase in myometrial contractility coupled with ripening of the cervix and activation of the fetal membranes and decidua (3, 12).

The immune system is hypothesized to play a key role in regulating the above processes, and a compelling body of evidence exists to suggest that both term and preterm labor are characterized by significant pro-inflammatory changes in gestational tissues (12–14). One point of difference is that the inflammation identified in term labor is commonly thought to be lower in magnitude than that identified in PTB (3, 12). Labor-associated inflammatory changes are characterized by immunocyte infiltration and significant increases in the expression of interleukin (IL)-1β, IL-6, IL-8, monocyte chemoattractant protein (MCP)-1, and tumor necrosis factor (TNF)-α expression in the fetal membranes, cervix, amniotic fluid, and placenta. Expression of pro-inflammatory mediators is, in turn, hypothesized to result in (i) increases in prostaglandins, which promote uterine contractility; (ii) degradation of the chorioamnion extracellular matrix; and (iii) ripening of the cervix by matrix metalloproteinases and reduced expression of tissue metalloproteinase inhibitors (14).

A complex regulatory network involving Th1 (IL-12 polarized CD4^+^ T-cells characterized by interferon-γ expression), Th2 (IL-4 polarized CD4^+^ T-cells characterized by IL-4, IL-10, and IL-13 expression), Treg (CD4^+^CD25^+^ T-cells expressing the transcription factor FOXP3), Th17 (CD4^+^ T-cells that secrete IL-17a and IL-17f), and uterine natural killer cells is hypothesized to be of critical importance in the establishment and prolongation of pregnancy (13–15). This network, in turn, is susceptible to regulation by the pro- and anti-inflammatory actions of a host of endocrine factors including progesterone, estrogen, human chorionic gonadotrophin, and luteinizing hormone (14, 15).

There is also good evidence to suggest that pro-inflammatory signaling following activation of the innate immune system, in response to injury or infection, plays an important role in the premature initiation of labor (11). Infection is most commonly identified in the earliest of preterm deliveries, those occurring before 28 weeks of gestational age. Goldenberg et al. suggest that the 25–40% of preterm deliveries ascribed to intrauterine infection may be a minimum estimate due to potential culture and sample selection bias (11). Similarly, histologic chorioamnionitis, a hallmark of intrauterine infection, has been reported in nearly 70% of preterm deliveries between 20 and 24 weeks’ gestational age, but in only 16% of cases delivered at 34 weeks (16). Recent data indicate that intrauterine infection is frequently polymicrobial in nature. Although bacterial nucleic acids have been identified in chorioamnion from both term and very preterm deliveries, samples from very preterm deliveries have been found to contain the largest diversity and distribution of bacterial species (17). The microorganisms most commonly identified in cases of PTB include the *Ureaplasma* spp. (in particular, *Ureaplasma parvum*), *Mycoplasma hominis*, and the *Fusobacterium* spp. There is also increasing evidence for the importance of *Candida* spp. in intraamniotic infection (18–20) and much remains to be understood in relation to the potential for viral infection to play a role in the pathological processes that underpin PTB.

We have previously speculated that the development of efficacious therapies for infection-associated PTB is likely predicated on our ability to resolve both intrauterine infection and the concomitant intrauterine inflammation that results (21). However, we also suggest that any attempts to regulate intrauterine inflammation must be undertaken with caution; regulated expression of cytokines plays an important role in the development of a number of fetal organs, including the skin, lung, and brain. To this end, identifying the origins and nature of the pathological inflammation that accompanies intrauterine infection is an important step in our efforts to deliver targeting anti-inflammatory therapies.

There is a wealth of data available to describe the pro-inflammatory responses of placental, uterine, cervical, and chorioamnion tissues to intrauterine infection. The contributions of somatic fetal tissues to both intrauterine inflammation and the fetal inflammatory response syndrome (FIRS; defined on the basis of a cord blood plasma IL-6 concentration >11 pg/mL) associated with intrauterine infection are, in contrast, much less well characterized.

The remainder of this paper will focus on data provided by basic science and clinical studies to (i) describe the capacity of fetal tissues, in particular, the skin, lung, gastrointestinal tract to mount a pro-inflammatory reaction in response to stimulation of the innate immune system by microbial agonist; and (ii) comment on some of the evidence, to date, that suggests an important role for these fetal tissues in driving the intrauterine inflammatory response implicated in PTB and fetal injury.

## Capacity of Fetal Tissues to Mount a Response to Infection and Injury via the Innate Immune System

The innate immune system is a critical element of host defense against microbial invasion. Broadly speaking, the innate immune system comprises humoral (endogenous antibodies and complement) and cellular (recognition of conserved pathogen motifs by natural killer cells, monocytes, macrophages, and non-immune cells including endothelial cells and neuronal cells, stimulating phagocytosis and or the release of pro-inflammatory cytokines, chemokines, and defensins) elements (22). Together, these act in concert to recognize and resolve infection in addition to serving as a means of coupling innate immune responses to the induction and amplification of a subsequent adaptive immune system response. A detailed treatment of the innate immune system is beyond the scope of this work and a number of excellent review articles have recently dealt with elements of the innate immune system including complement (23), toll-like receptors (TLRs) (24), Nod-like receptors (25), and defensins (26). Rather, this work will take three key elements of the innate immune system, namely, pattern-recognition receptors, complement, and defensins, and explore the evidence for their expression in developing fetal tissues.

## Pattern-Recognition Receptors

Pattern-recognition receptors (PRRs) are innate immune system receptors that recognize structurally stable microbial elements (27). In addition, PRRs can recognize molecules that are released in response to tissue injury or stress (24, 27). Their immunological role is considered to be of great importance as their conserved ability to recognize pathogens and/or injury allows for an initial defensive reaction, which simultaneously recruits elements of the adaptive immune system to respond to a potential infectious threat. PRR ligand binding results in the activation of multiple signaling factors (including NF-κB and interferon-regulatory factors), yielding cytokine and chemokine expression, with down-stream effects including enhanced phagocytosis, pyrexia, increased rates of hematopoiesis, elevated type-I interferon expression, and pyroptosis (27).

Toll-like receptors are a large family of transmembrane proteins, and perhaps the best characterized class of PRRs. TLRs 1–6, 10, and 11 are found in the plasma membrane and TLRs 3, 7, and 9 in endosomal membranes. While most TLRs have quite defined ligand targets (e.g., TLR 5 recognizes flagellin, TLR 7 recognizes single stranded RNA), TLR 2 and TLR 4 both recognize a wide range of ligands including high-mobility group box protein 1 (HMGB1), peptidoglycan, mucins, hemagglutinin, and porins for TLR 2; and lipopolysaccharide (LPS), vesicular stomatitis virus glycoprotein G, mannan, heat shock protein (HSP) 60, HSP 70, and HMGB1 for TLR 4 (24).

Studies in human fetal autopsy samples have detected the presence of TLRs from an early gestational age. Immunohistochemical studies have demonstrated TLR 3 expression in neuronal and glial cells in preterm brains from approximately 23 weeks’ gestational age (28). Petrikin and colleagues used a PCR array platform validated by hydrolysis probe qPCR to analyze the temporal expression of TLRs in the human fetal lung at 60-, 90-, and 130-day gestational age. TLRs 1–8 and 10 were identified in lung samples from 60-day gestational age fetuses, indicating that TLR signaling is likely functional from very early in fetal development. Interestingly, with the exception of TLR 4, the expression of all TLR transcripts increased significantly (between 1.43- and 9.20-fold) from 60 to 130 days of gestation (29). Flow cytometry studies have demonstrated that TLR4 is expressed on the surface of CD14^+^ monocytes harvested from preterm infants delivered at <30 weeks’ gestational age, although at levels significantly lower than that seen in preterm infants born later than 30 weeks’ gestation. A similar pattern was identified for TLR4 mRNA transcripts in CD14^+^ monocytes (30). Increasing expression of TLR 2 in the lung with gestational age was identified in studies of fetal ovine TLR expression between 108 and 145-day gestational age (31). Again using qPCR, Sow et al. demonstrated the presence of mRNA transcripts for TLRs 4, 5, 7, and 8 at 115-day gestational age in the fetal ovine lung (32).

Toll-like receptor expression has also been characterized in the fetal ovine skin and spleen across the second trimester of pregnancy; work by Nalubamba and co-workers suggests that TLRs 1–10 are expressed in the spleen at levels approaching or often exceeding that seen in the adult from 60 days of gestation (33). TLR expression in the fetal skin was also found to be extensive, although transcripts for TLRs 9 and 10 were not identified between 60 and 90 days of gestation (33). A recent study by Iram and colleagues reported that skin samples taken from embryonic (9–11 weeks’ gestation) to fetal (12–13 weeks’ gestation) autopsies expressed the same range of TLRs as adult skin. Their data also suggested that, mRNA transcripts for TLRs 1–5 were more highly expressed in these early gestation skin samples than in adult skin samples (34).

In summary, these studies provide strong evidence to suggest that in the developing fetus: (i) TLRs are expressed from very early in gestational development; (ii) TLR expression is widespread in both AF-exposed (lung, skin) and internal (brain, spleen, immunocytes) fetal tissues; and (iii) the ontological pattern of TLR expression likely varies between tissues, which, in turn, potentially impacts the ability of individual tissues to mount a response to tissue invasion and injury by microorganisms.

In the cytosol, retinoic acid-inducible gene 1 (RIG-1)-like helicases, including RIG-1 and melanoma differentiation-associated gene 5 (MDA5), detect double stranded viral RNA and play an important role in the regulation of interferon expression in response to viral infection (35, 36). Work in embryonic chickens indicates that MDA5 is expressed in both the fetal spleen and lung from at least embryonic day 10 (35). In studies utilizing primary fetal buffalo fibroblast cells, Poly I:C treatment resulted in significant increases in RIG-1, MDA-5, and interferon-β mRNA expression, suggesting that the presence of a functional innate immune response to simulated viral infection (36). RIG-1, MDA5, and interferon-β1 mRNA transcripts have also been shown to be up-regulated in primary human cord blood mast cells in response to stimulation with antibody enhanced dengue virus infection (37). NOD-like receptors are a third class of PRRs; NOD-1 and NOD-2 recognize specific peptidoglycan derivatives (meso-diamino-pimelic acid and muramyl dipeptide, respectively) to initiate pro-inflammatory NF-κB and MAP kinase signaling (38). Several other NOD-like receptors are implicated in multiple immunomodulatory roles including inflammasome function and type-I interferon production (27). A recent analysis of NOD-like receptor Nlrp6 inflammasome elements by Kempster and colleagues demonstrated that pycard, caspase-1, and IL-18 (an inflammasome substrate) mRNA transcripts are detectable in the fetal ovine jejunum at 100-day gestational age. In the same study, Nlrp6, pycard, and caspase-2 transcripts were detected in the fetal rat intestine and lung at both embryonic day 16 and 20 (39).

Considered together, these data from animal and human studies suggest that functional TLR, RIG-1-like helicase, and Nod-like receptor PRR systems are widely expressed from early in pregnancy (Table 1). As such, it appears reasonable to conclude that these innate immune system effectors are capable of playing a role in the fetal response to intrauterine infection.

**Table 1:** **Summary of pattern-recognition receptor expression in human and animal fetal tissues**.

PRR class/type	Fetal studies			Reference
	Component	Organism and tissue	Fetal gestational age (days)	
Toll-like receptors	TLR3	Human neuronal and glial cells	161	(28)
	TLRs 1–8 and 10	Human lung	60	(29)
	TLR2	Ovine lung	108	(31)
	TLRs 4, 5, 7, and 8	Ovine lung	115	(32)
	TLRs 1–10	Ovine spleen	60	(33)
	TLRs 1–5	Human skin	84	(34)
RIG-1-like receptors	MDA5	Avian spleen and lung	10	(35)
NOD-like receptors	Pycard, caspase-1, IL-18	Ovine jejunum	100	(39)
	Nlrp6, Pycard, caspase-2	Rodent intestine and lung	16	(39)

## Complement

The complement system is a central component of the humoral innate immune system (40). To date, three pathways of complement activation have been identified (i) the classical pathway, which is activated in response to antigen–antibody complex formation; (ii) the constitutively active, antibody–antigen complex independent alternative pathway; and (iii) a more recently identified lectin pathway, which is activated following the formation of complexes between mannin binding lectin and surface expressed microbial mannose (41). Cleavage of C3 into C3a and C3b is a key step in complement activation common to all three activation pathways. C3b is a multifunctional complement mediator; it binds foreign cells, identifying them for phagocytosis. In addition, C3b interacts with C5 to initiate a cleavage cascade that results in the formation of a lytic membrane attack complex that damages the membrane integrity of foreign cells (41). Complement proteins also have a range of innate immunomodulatory functions (42); for example, C5a and C3a act as chemoattractants via interactions with CD88 and C5L2 receptors, recruiting immunocytes (including lymphocytes and neutrophils) to sites of microbial invasion or localized inflammation (40).

Although much remains to be understood in relation to the ontogeny of fetal complement expression, there are a number of clinical studies that demonstrate elements of the complement system are expressed from early in gestation (40, 43). At term, serum complement factors’ concentrations have been reported to range from 36 to 79% of normal adult levels (44). As summarized by Grumach et al., complement pathway components are detectable as early as 5 weeks’ gestation. The authors note that (p. 267), “one may assume that all the components may be detected by week 18–20 of pregnancy” (43). CH_50_ (a sheep erythrocyte-based lysis assay to determine the functional activity of the classical and terminal complement pathways) studies with preterm infants suggest that functional complement activity at 27–31 weeks of gestation is approximately one-third of the normal adult level (40, 45). Woolach and colleagues reported an AP_50_ (a rabbit erythrocyte-based lysis assay that employs a calcium chelator to inactivate the classical and lectin pathways to isolate alternative pathway functional activity) value of approximately 50% normal adult levels for preterm infants born between 28 and 33 weeks’ gestation (44). A similar pattern is apparent in fetal sheep. Analysis of data from modified CH_50_ assays using serum from fetuses delivered at 125-day (term is between 145 and 150 days) revealed a pattern of lytic activity similar to that of maternal sheep serum. In contrast, maximum lysis using serum from earlier gestational ages (85 and 95 days) was approximately twofold lower (46).

Together, these data suggest that the fetus is equipped with a functional complement system from early in gestation, although it is active at lower levels than in adults.

## Defensins

Defensins are a class of antimicrobial proteins that are released from a variety of cell types in response to the detection of tissue injury or microbial pathogens. They are found at highest concentrations in cells that play a role in host defense including leukocytes (where they are stored in granules) and Paneth cells in the small intestine. Defensins are also produced by a range of epithelial cells, either constitutively or following stimulation by microbial agonist (47). Human beings have six α-defensins (HαD) and at least 28 β-defensins (HβD), although only HβD 1–6 and 23 have been well characterized (26). HαD 1–4 are commonly termed human neutrophil peptide (HNP) 1–4 due to their constitutive synthesis by neutrophil precursors in bone marrow (47). HαD 5 and 6 are commonly termed human defensins (HD) 5 and 6. They are expressed by intestinal cells in addition to a number of types of epithelial cells. In mammals, epithelial cells are the primary sources of HβDs (26).

As elegantly reviewed by Yang and colleagues, defensins play a multitude roles in host defense. Both HαDs and HβDs exhibit differential microbiocidal activity by the formation of pore-like structures in microbial cell membranes, resulting in membrane disruption and depolarization (47). HβD 1 and HβD 2, for example, are effective at killing *Escherichia coli* and *Pseudomonas aeruginosa* but lack HβD 3’s ability to kill *Staphylococcus aureus* (26). Defensins have also been shown to have anti-viral activity, can neutralize microbial toxins, and act as both chemoattractants and activators of phagocytic activity. Importantly, they have also been shown to mobilize dendritic cells and T-lymphocytes, bridging the innate and adaptive immune responses to combat infection (26). Given their importance to host microbial defense, a number of investigators have undertaken studies in human beings and animals in an attempt to clarify the ontological development of fetal defensin expression across gestation.

A mounting body of evidence exists to suggest that defensins are expressed early in gestation across a wide range of organisms. Isoforms of the β-defensin-like gene have been identified in an expressed sequence tag library derived from the early developmental stages of the olive flounder, *Paralichthys olivaceus* (48). Working with fertilized chick eggs over a 12-day time course, Meade and co-workers demonstrated the expression of 13 avian β-defensins at 3-day post-laying. Interestingly, differing patterns of both temporal and spatial (head vs. abdomen) expression were evident in the magnitude of avian β-defensin expression over the 12-day time course, suggesting that a process of developmental regulation with embryonic development (49). Data from a more recent histological study using immunolabelling suggest that peridermal granules in the developing embryonic chick skin contain avian β-defensin 9, indicating that these organelles play a role in both host defense and epidermal barrier formation (50). In sheep fetuses, initial work by Huttner et al. demonstrated the presence of ovine βD1 and βD2 mRNA transcripts in jejunum, ileum, caecum, and colon of 130-day gestational age fetuses (51). These findings were later replicated, in part, by work undertaken in late gestation (120–140-day gestational age) sheep fetuses, with Meyerholz and colleagues reporting the identification of ovine βD2 expression in both the small and large intestine (52).

Data from human fetal studies also suggest that a pattern of spatial and temporal regulation for defensins. Using semi-quantitiative PCR, Starner et al. demonstrated that HβD1, HβD2, but not HβD3 were expressed in the fetal lung at 42 weeks’ gestational age; all three HβDs were undetectable at 18 and 22 weeks of gestation. Interestingly, the human cathelicidin, LL-37, was found to be expressed at all three gestational time points, with the highest apparent expression at 18 weeks’ gestation (53). A distinct pattern of defensin expression is also apparent in the fetal skin. Immunohistochemical studies have shown that HβD2 and HβD3 staining is absent from the fetal skin at 10–15 weeks of gestational age, and that HβD3 is expressed in the *stratum corneum* at 18–24 weeks’ gestation (54). *In vitro* studies with primary human fetal keratinocytes suggest that HβD1 and HβD2 mRNA is often more highly expressed at 22 weeks’ gestation than in samples from neonatal and adult skin (55). Defensin proteins have also been identified in human amniotic fluid and *vernix caesosa* samples from term pregnancies without chorioamnionitis and in the amniotic fluid of women with preterm labor (56, 57).

## Fetus, Infection, and Intrauterine Inflammation

Fetal inflammation is strongly associated with impending preterm labor, preterm prelabor rupture of membranes (PPROM) and is also an independent risk factor for subsequent neonatal morbidity (58). Elevated levels of cord blood IL-6 at birth have, for example, been demonstrated to predict the development of bronchopulmonary dysplasia (59). In a now classic study, Gomez and colleagues proposed that a fetal cord blood plasma IL-6 concentration in excess of 11 pg/mL was indicative of a FIRS, a condition that the authors describe as being (p. 201) “frequently associated with microbial invasion of the amniotic cavity” (58).

Intrauterine infection is associated with chorioamnionitis and funisitis, which are, in turn, inversely related to gestational age at delivery (16, 60). To further investigate the relationship between intrauterine infection and fetal inflammation, Yoon and colleagues examined putative relationships between umbilical cord blood IL-6 concentration, funisitis, amniotic-fluid microbial culture, and neonatal sepsis in a cohort of 315 preterm (20–35 weeks’ gestation) infants. The presence of oligohydramnios, which is associated with a range of neonatal morbidities, has also been associated with elevated cord blood IL-6 concentrations in women with PPROM (61). Preterm infants with funisitis were found to have elevated rates of chorioamnionitis and positive amniotic-fluid cultures, a lower gestational age at delivery, and higher cord blood IL-6 concentrations than those without funisitis. These data suggest that funisitis is also associated with a FIRS (60). Fetal plasma IL-6 levels have also been shown to be significantly associated with inflammatory lesions in the chorioamnion, leading to the conclusion that funisitis and chorioamnionitis are histological markers of FIRS (62).

Interestingly, subsequent work by Lee et al. demonstrated that, in an analysis of patients with intrauterine inflammation (defined on the basis of an elevated matrix metalloproteinase 8 concentration in the amniotic fluid), cord blood plasma C-reactive protein was lower in the absence of proven amniotic-fluid infection, compared to cases where infection was confirmed by culture. These data suggest that although FIRS is possible in the absence of intraamniotic infection, the strongest fetal inflammatory response is associated with a culturable amniotic infection (63). Recent studies suggest that rather than having a simple binary relationship, it is important to take into account the magnitude of amniotic-fluid inflammation when predicting pregnancy outcomes (64). On the basis of these data, it is also tempting to speculate that a similar pattern may be applicable with regards to cord blood plasma IL-6 levels and the severity of FIRS.

Much remains to be understood regarding the differential impact of intraamniotic infection with differing microorganisms (either in isolation or simultaneously with one or more other microorganisms) on the systemic fetal inflammatory response; Lee and colleagues, for example, noted the isolation of some 11 species of microorganisms from the amniotic cavity and identified polymicrobial infections in 6 out of 89 cases (63). Similar findings suggesting that the involvement of a wide range of microbial species and a significant proportion of polymicrobial infections of the amniotic environment have been reported by both DiGiulio and Jones (17, 65).

How the presence of multiple immunomodulatory microbial agonists alters fetal inflammatory signaling *in utero* is still imprecisely understood. Based on the observations that (i) intrauterine inflammation is elevated in preterm compared to term labor; (ii) culture positive amniotic-fluid infection is associated with more severe fetal inflammatory responses; and (iii) polymicrobial infections are more common in labor at earlier gestations, one might be tempted to conclude that a polymicrobial infection would equate to a more severe inflammatory response. However, data, to date, suggest that the intrauterine inflammatory response is likely influenced by (at least) the magnitude and timing of microbial agonist exposure. The immunomodulatory effects of one microorganism, perhaps by down-regulating antimicrobial peptide expression as has been shown by *Ureaplasma* spp., may allow for additional microorganisms to establish *in utero* (66). It is also possible that the pathogen-specific inflammatory responses of different microorganisms combine to trigger multiple signaling pathways required to initiate preterm labor (67).

Findings from *in vitro* studies with preterm and term human monocytes, for example, suggested that *Ureaplasma urealyticum* exposure modified the pro-inflammatory response to a subsequent stimulation with *E. coli* LPS over a 24 h time course, and did so in a dose-dependent fashion. Exposure to a high dose (10^6^ color change units) of *U. urealyticum* enhanced the release of TNF-α and IL-8, but not IL-6 and IL-10 (68). The authors speculated that the basis of the enhanced pro-inflammatory response was due to the differential suppression of regulatory cytokine (e.g., IL-10) expression. Evidence of an interaction between *U. parvum* and LPS is also provided by work in a sheep model of intrauterine infection. Chronic (70 days) but not acute (7 days) *U. parvum* exposure has been shown to inhibit pro-inflammatory signaling in the fetal lung (69). Surprisingly, acute *U. parvum* exposure was subsequently reported to have an LPS-inhibitory effect in the chorioamnion, suggesting that agonist, exposure timing, and tissue all impact inflammation deriving from intrauterine infection (70).

Data from clinical studies that suggest a fetal inflammatory response to direct stimulation by microbial agonist are supported by a number of studies undertaken in non-human primate, sheep, rabbit, and rodent models of human pregnancy. With the ability to control dose, type of exposure, and length of exposure, animal models of intrauterine infection have been important in advancing our understanding of the fetal inflammatory response to different microbial agonists.

Working with an ascending model of intrauterine infection in rabbits, Yoon et al. demonstrated that cervical inoculation of 10^3^–10^4^ colony forming units of *E. coli* resulted in white matter injury in pups euthanized 5–6 days after inoculation (71). Subsequent studies in a similar model system investigated the acute intrauterine responses to *E. coli* intrauteruine infection. Over a 30 h time course, progressive histologic inflammation was identified in the uterus, placenta, and lung (72). Interestingly, although mitotic activity in the pup brain was found to be retarded after 8 h post-inoculation, there was no evidence of brain inflammation or apoptosis suggesting that a potential lag in the transduction of inflammation from an infection of the amniotic fluid. The effects of intraamniotic *E. coli* LPS exposure have also been extensively characterized in the sheep using ultrasound-guided intraamniotic injections. LPS exposure has been shown to result in time and dose-dependent inflammatory responses in the chorioamnion, systemic fetal inflammation, changes in fetal lung development, and fetal brain inflammation (73–77).

Although *E. coli* and *E. coli* LPS serve as useful reagents for studying intrauterine inflammatory responses, *E. coli* infection in the setting of preterm delivery is comparatively uncommon. In contrast, the *Ureaplasma* spp., and especially *U. parvum*, are among the microorganisms most commonly identified by culture or PCR in cases of PTB. Intraamniotic inoculation of chronically catheterized macaques with clinical isolates of *U. parvum* serovar 1 or *M. hominis* resulted in significant increases in amniotic-fluid leukocytes within 24 h and amniotic-fluid TNF-α, IL-1β, Il-6, IL-8 between 48 and 72 h post-inoculation (78). Studies with group B streptococci in similarly catheterized macaques revealed increases in amniotic-fluid IL-1α and IL-6 at 15–18 h post inoculation, substantially earlier than that seen in response to intrauterine infection with *U. parvum*, suggesting that a differential inflammatory response to microbial agonist (78). Studies undertaken in the chronically infected sheep further underscore the pro-inflammatory role *U. parvum* in pregnancy; intrauterine *U. parvum* infection has been shown to be associated with a progressive chorioamnionitis, changes in fetal growth and fetal inflammation (79–81).

As summarized above, there is now ample evidence from clinical and animal studies to demonstrate that infection of the uterus is associated with intrauterine inflammation, and that the fetus is well equipped to respond to such infection with a robust pro-inflammatory response. Accordingly, it is of interest to determine the origins of this intrauterine inflammatory process in order to assist in our understanding of the pathological processes underpinning infection-associated PTB and our attempts to develop targeted interventions.

In many cases, it appears that fetal bacteremia is less common than colonization or infection of gestational tissues such as the placenta or the amniotic fluid. Recent studies in sheep, for example, demonstrated that fetal *U. parvum* bacteremia is uncommon despite the presence of viable microorganisms in the amniotic fluid (82). It is unclear, however, whether fastidious microorganisms such as *U. parvum* have the ability to replicate in amniotic fluid or if they are seeded into it following growth on amniotic-fluid exposed tissues.

A study investigating the acute fetal responses to intraamniotic *Candida albicans* infection demonstrated an absence of fungal RNA in the fetal ovine spleen in conjunction with positive amniotic-fluid cultures and the detection of *C. albicans* RNA in the fetal lung and skin (20). Similarly, in findings from the Alabama preterm birth study, placental cultures for *M. hominis* and/or *U. parvum* were positive in 63.4% of male fetuses. In contrast, only 27.6% of fetuses had positive cord blood cultures (83). For future studies, it will be of particular interest to determine how modifiable factors including gestational age, length of infection, and the infectious organism itself interact to influence the prevalence of fetal bacteremia in infection-associated PTB.

There is also evidence to suggest that pro-inflammatory microbial agonists in the amniotic fluid, such as LPS, does not passively diffuse across cell layers (84). These findings go some way to explaining the observation that while 1 μg/kg is considered a sub-lethal intravenous bolus dose of *E. coli* LPS in the sheep fetus, it is possible to inject in excess of 10 mg *E. coli* LPS into the amniotic fluid of pregnant ewes without causing fetal death (74, 85, 86). Interestingly, the intraamniotic administration of LPS from other Gram negative microorganisms associated with PTB has been shown to have a much more severe impact on fetal wellbeing, although it is unclear if this relates to a more toxic inflammatory response or enhanced ability to penetrate the fetal circulation. Studies in a sheep model of pregnancy, for example, have shown that *Porphyromonas gingivalis* LPS exposure results in much higher rates of fetal death than *E. coli* LPS (87). A recent analysis of human fetal membrane responses to PTB-associated microorganisms provides further evidence for an organism-specific and host-specific inflammatory effects deriving from the presence of microorganisms in the amniotic fluid. Peltier and colleagues demonstrated that PTB-associated pathogens including *E. coli*, *Gardnerella vaginalis*, Group B streptococci, and *Ureaplasma* spp. elicited differing pro-inflammatory responses in human fetal membrane explant studies. For example, *G. vaginalis* stimulated IL-1β and TNF-α production, whereas *U. parvum* elicited IL-10 and TNF-α expression but had no effect on the concentration of IL-1β (88). These data suggest that tissues directly exposed to the amniotic fluid are likely key in the propagation of amniotic-fluid inflammation in response to intrauterine infection and likely also contribute to systemic fetal inflammation. With this in mind, the remainder of this paper will discuss evidence for the contribution of the fetal lung, skin, and gastrointestinal tract to intrauterine inflammation.

## Contribution of Fetal Lung, Skin, and Gastrointestinal Tract to Intrauterine Inflammation

Inflammation in the fetal lung in response to microorganisms in the amniotic environment has been a subject of significant interest for several decades and the primary focus of fetal inflammation in association with PTB (89). Broadly speaking, work in this area may be classified into two broad avenues of investigation: (i) the localized impact of intrauterine infection on the lung itself, including structural (90, 91), functional (92), and inflammatory adaptations (73, 76, 93); and (ii) the extra-pulmonary effects of inflammation in the fetal lung, including changes in fetal systemic inflammation (94–96), immunocyte reactivity (97–99), and amniotic-fluid inflammation (100).

The fetal lung has been shown to generate a pro-inflammatory response following exposure to a wide range of microbial agents including adenovirus (101, 102), *E. coli* LPS (73, 103, 104), *Ureaplasma* spp. (81, 105), and *C. albicans* (20), which is in keeping with studies suggesting that the developing fetal lung expresses a wide range of PRRs from early in gestation (29). Human lung cells have also been shown to express complement factors, but not defensins, in response to endotoxin exposure (103, 106). Infection with *C. albicans* elicits an exceptionally vigorous inflammatory response in the fetal lung, but a much milder response in the chorioamnion (20). In comparison, the response elicited by *Ureaplasma* spp. is much more mild with to regards fetal lung inflammation (105). It is also hypothesized that signaling by pro-inflammatory cytokines plays a key role in driving lung maturation. Evidence in support of this model have been provided by studies in the sheep, wherein LPS-driven inflammation in the chorioamnion (occurring as early as 5 h after intraamniotic LPS injection) and lung (occurring 1–2 days after intraamniotic LPS injection) preceded lung maturation by as much as 5 days.

A number of cytokines have also been shown to exert differential effects on the fetal lung. Increases in a range of cytokines including IL-1, IL-6, IL-8, and TNF-α in the amniotic fluid and fetal lung are associated with intrauterine infection and preterm labor (107). Interestingly, work in a sheep model of pregnancy demonstrated that intraamniotic IL-8 exposure resulted in only small increases in bronchoalveolar lavage immunocytes and did not induce lung maturation (108). A similarly small response in the lung was identified following exposure to TNF-α (109).

In contrast, IL-1α and IL-1β both elicit marked pro-inflammatory and maturational effects on the fetal ovine lung, with IL-1α driving the strongest response (110). IL-1α has also been shown to elicit a dose-dependent increase in surfactant protein-A and protein-B mRNA expression in rabbits (111). Subsequent work by Sosenko et al. suggested that the pulmonary effects identified in association with intraamniotic IL-1α result from direct contact with the fetal lung (112). These observations may hint at a potential role for the lung in amplifying IL-1 inflammatory responses derived from the fetal membranes, skin, or gut, as well as acting as a transducer of intraamniotic inflammation to systemic fetal inflammation.

The role played by the lung in contributing to extra-pulmonary inflammation is of particular interest given the strong association between FIRS, PTB, and neonatal morbidities including bronchopulmonary dysplasia and white matter injury. Between 85 and 115 days’ gestation, the ovine fetal lung produces lung fluid at a rate of 2–3 mL/kg/h and it is estimated that 50% of that volume is excreted into the amniotic fluid with a mixing time of 2–3 h (113, 114). As noted in seminal work by Tomoda et al., the fetus is estimated to swallow amniotic fluid at a substantial rate, with reported daily volumes varying between 200 and 1000 mL (115). In fetal sheep, *E. coli* LPS has been reportedly detected in the fetal stomach 2 days after intraamniotic exposure (116). As such, it seems likely that pro-inflammatory mediators excreted by the pulmonary epithelium would rapidly make their way into the amniotic fluid, where they could interact with immunocytes in the amniotic fluid, the fetal skin and membranes, and, after swallowing, the fetal gastrointestinal epithelium. Although the relative contributions of tissues amniotic-fluid inflammation remain unclear, it seems likely that the lung is an important source of inflammatory cytokines and white blood cells. Using polymyxin-B to bind and inhibit the inflammatory activity of intraamniotic *E. coli* LPS, Saito and colleagues demonstrated that a reduction in fetal lung mRNA expression was associated with significant reductions in cytokine protein concentration in the amniotic fluid (100).

In clinical studies, elevated white blood cell counts in the amniotic fluid are associated with intrauterine infection and an increased risk of spontaneous preterm delivery (117, 118). A significant majority of the leukocytes found in the amniotic fluid in association with intrauterine inflammation are of fetal origin (119). It is tempting to speculate that the lung is a likely source of leukocytes in the amniotic fluid, although it is possible that cells migrate across the gastrointestinal epithelium and developing skin in response to a chemotactic gradient. Indeed, Payne and colleagues have recently demonstrated the presence of clusters of basophilic cells in the apical layers of the developing fetal skin in response to experimental *C. albicans* infection (20). In human neonates, prolonged rupture of membranes during pregnancy has been associated with elevated white blood cells and IL-6 in bronchoalveloar lavage samples taken within 24 h of delivery (120). Similarly, infants with funisitis born prior to 28 weeks’ gestation were found to have higher levels of CD68^+^ cells in tracheobronchial aspirate fluid taken within 24 h of delivery than those without funisitis (121). Sheep infected with intraamniotic *U. parvum* serovar 3 were also shown to have statistically significant increases in bronchoalveolar neutrophils after 3 days of exposure (105).

Although the lung has been the major focus of fetal inflammatory responses to intrauterine inflammation, there is evidence to suggest that the fetal skin and gastrointestinal tract also generate a pro-inflammatory response following exposure to microbial agonists. In a fetal autopsy study, Kim and colleagues demonstrated fetal skin inflammation and TLR-2 regulation in association with microbial invasion of the amniotic cavity, concluding that dermatitis is a component of the FIRS (122). Subsequent studies in sheep have demonstrated that the intraamniotic injection of either *E. coli* LPS, *U. parvum* serovar 3 or *C. albicans* results in a pro-inflammatory response in the skin, with *C. albicans* eliciting the strongest response (20, 74, 79, 123). T-cells (present in the second trimester), mast cells (present from 12 weeks’ estimated gestation), and antigen presenting cells (present from at least 9 weeks’ estimated gestation) have been identified in the developing human dermis from early in fetal life (124). Fetal keratincoytes have been shown to respond directly to stimulation with microbial agonist and are known to express a wide range of antimicrobial peptides (55, 100, 125, 126). In the developing fetal gut, endotoxin exposure in preterm sheep is associated with disrupted structural development of the intestinal epithelium. Although an inflammatory response was not evident at 2 days after endotoxin administration, significant infiltrations of T-lymphocytes and myeloperoxidase cells were detected at 14 days-post exposure (116). These data suggest that a temporally different response in the fetal gut to inflammatory agonist when compared to more acute responses identified in the fetal lung and skin. Subsequent work, again in the fetal sheep, has suggested that a critical role for IL-1α in these gut-modifying processes, reminiscent of earlier data showing a similar role for IL-1α in the fetal ovine lung (110, 127).

With regards to systemic fetal inflammation, the available data suggest that the lung is likely a key mediator of transducing inflammatory signaling from the amniotic fluid to the internal fetal organs and the fetal gastrointestinal tract (94, 96, 128). Although inflammation of the fetal lung has been demonstrated to induce systemic responses and cause inflammation in the fetal gut, exposure of the fetal skin and amnion to similar stimulation did not result in detectable inflammation in the lung or the gut (94, 96). However, it does appear that both the fetal skin and fetal gut play a smaller contributing role to the overall induction of FIRS (94, 128). Kramer et al. demonstrated a differential systemic fetal inflammatory response to acute *E. coli* LPS exposure, which was dependent on the fetal organs stimulated with agonist (128). Kemp and colleagues subsequently demonstrated that acutely exposing the fetal lung to *E. coli* LPS resulted in increased IL1-β, IL-6, and IL-8 mRNA in the fetal spleen, increased cord blood plasma MCP-1, and elevated cord blood white blood cell counts. Smaller changes were identified in response to fetal skin and amnion exposure, but not in response to acute gut exposure (94, 96). These data are also in keeping with previous studies that suggest a longer inflammatory response time for the fetal gastrointestinal tract compared to the skin or lung. Underscoring the complexity of intrauterine inflammatory signaling, these data also point to a differential temporal and spatial response to fetal inflammatory stimulation that likely varies with the microbial agonist in question.

## Conclusion

As a function of PTB, the contribution of the fetus to intrauterine inflammation and FIRS is complex and, based on the evidence presented herein, likely dependent on a wide range of modifying factors. Evidence is available to suggest that gestational age at the time of infection, the nature of the infection itself (single organism vs. polymicrobial infection), the host’s genetic background in addition to differences in specific tissue responses each combine to impact the origins and development of the intrauterine inflammation associated with PTB. It is clear, however, that the fetus is well endowed with the immunological armamentarium necessary to respond to microbial invasion of the amniotic fluid. Moreover, we now know that the fetus possesses the ability to recognize and respond to microbial agonist via elements of the innate immune system from comparatively early in gestation.

There is a compelling body of evidence from basic science and clinical studies to demonstrate that intrauterine infection is strongly associated with fetal inflammation. There is also excellent data to suggest that the intrauterine inflammation that is implicated in the precocious initiation of labor also plays a role in the development of a number of the congenital pathologies that are commonly identified in preterm infants, such as bronchopulmonary dysplasia and white matter injury. Accordingly, identifying the spatio-temporal origins of intrauterine inflammation, and how it might differ on an individual, case-by-case basis, is likely an important requirement in our efforts to develop treatments that prevent PTB while ensuring the continued development of a healthy fetus.

## Conflict of Interest Statement

The author declares that the research was conducted in the absence of any commercial or financial relationships that could be construed as a potential conflict of interest.
